# Teleassessment can overestimate the risk of learning disability in first and second grade of primary school

**DOI:** 10.1186/s13052-025-01881-4

**Published:** 2025-02-11

**Authors:** Stefania Fontolan, Sandro Franceschini, Marisa Bortolozzo, Linda Greta Dui, Simona Ferrante, Cristiano Termine

**Affiliations:** 1 Department of Medicine and Technological Innovation, University of Insubria, Via Guicciardini 9, Varese, 21100 Italy; 2 Department of Medicine and Surgery, University of Insubria, Via Guicciardini, 9, 21100 Varese, Italy; 3 NearLab, Politecnico di Milano, Department of Electronics, Information and Bioengineering, Via Colombo 40, 20133 Milan, Italy; 4 LEARNLab, Joint Research Platform, Fondazione IRCCS Istituto Neurologico Carlo Besta, Via Celoria 11, 20133 Milan, Italy

**Keywords:** Evaluation methodologies, Remote assessment, Learning difficulties, Primary school, Test modality comparison

## Abstract

**Background:**

Early administration of reading, writing and math standardised tests allows us to assess the risk of developing a learning disorder and to plan a specific intervention. The ease of access to technological tools and past pandemic restrictions have led to the abandonment of face-to-face assessment in favour of teleassessment methods. Although these kinds of assessments sometimes seem comparable in the literature, their equivalence is not clearly defined. The first aim of our research was to test the comparability of the two modalities using a complete battery of neuropsychological tests. Second, we addressed whether the administration order could influence performance.

**Methods:**

Using a within-subject sample design, we compared face-to-face and teleassessment performance in reading, writing and math tasks in 64 children attending first and second year of primary school.

**Results:**

Teleassessment scores were lower than face-to-face; math tests weighted on difference. Differences were mitigated by previous experience with face-to-face modality.

**Conclusions:**

Although there was considerable overlap between the two administration methods, teleassessment could lead to overestimation of the risk for learning disorders.

**Supplementary Information:**

The online version contains supplementary material available at 10.1186/s13052-025-01881-4.

## Introduction

### The importance of early screening in the learning domain

Learning disorders are among the most frequently diagnosed developmental disorders in childhood and are characterised by difficulty in the acquisition and use of school skills in the domains of reading, writing, and math.

Languages with transparent orthography allow faster acquisition of learning abilities [1, 2]. Due to the rapid acquisition of basic school abilities, through the administration of reading, writing, and math standardised tests in the first two years of primary school, it is possible to assess whether performance falls within the normal range.

Far from being a complete clinical evaluation, a first screening—conducted by psychologists, neuropsychiatrists or trained teachers—during the first two years of primary school ensures quick access to supplementary didactic activities or targeted interventions for all children with learning difficulties [3–6]. The possibility of administering a teleassessment evaluation could represent an opportunity to guarantee a faster connection between school and clinical services.

### Neuropsychological teleassessment

The ease of access to technological tools observed in recent decades and during the spread of the COVID-19 pandemic has given a strong impetus towards the abandonment of face-to-face assessment methods and has favoured the use of teleassessment modalities [7–9].

Neuropsychological tests are usually administered in a face-to-face condition, and this is the modality in which tests are usually designed and standardised. However, the spread of technology and the possibility of conducting tests in different modalities enable us to study the influence of administration modality on performance.

Krach et al. [10, 11] pointed out that although the equivalence of the outcomes of paper-and-pencil and computerised assessments has been demonstrated for some tests, often in both assessment conditions, the taker and test administrator were in the same room. The control of this variable appears to be of primary importance, considering that isolation, and consequently the physical distance between the administrator and the taker, falls within the definition of teleassessment [12, 13].

Teleassessment can be defined as a diagnostic psychological assessment procedure (in real time or at different time points) administered to individuals who are not in the same room as the examiner using telecommunication technologies [10, 11]. Nevertheless, in the case of children’s teleassessment, the presence of a passive operator, who can support the child in the use of materials, is often useful.

The main strengths of teleassessment include the possibility of (a) accessing geographic areas far from clinical centers and (b) enabling early diagnosis. Negative influences on teleassessment evaluation could be induced by a slow internet connection, which can prevent a flowing dialogue, and by low-quality devices, which can make it difficult to show task stimuli on the screen. Nevertheless, teleassessment appears to be related to a good level of satisfaction among participants, and the use of information and communication technologies for the evaluation of cognitive skills is thought to provide a motivating environment that could increase compliance [14, 15].

Multiple tests that are useful for measuring cognitive skills such as short- and long-term memory, visual matching, processing speed, and logical reasoning in school-aged children [e.g. 16, 17] have been developed for computer devices, avoiding the use of paper-and-pencil materials; for this reason, they appear suitable for teleassessment. Many other tests, from simple interviews to the assessment of complex cognitive functions, could be easily administered in teleassessment modality with minimal changes to the original face-to-face test [e.g. 18, 19, 20]. Nevertheless, it has not yet been determined whether face-to-face assessment and teleassessment provide an equivalent evaluation: changes in material or distance from the administrator could cause differences—especially in children—in engagement, attention, and compliance, with a cascade effect on final scores [7, 21]. To ensure equivalency between teleassessment and face-to-face instruments, multiple procedural arrangements or psychometric requirements are necessary [11, 22].

### Teleassessment of cognitive functions

In the cognitive domain, Wright [23], in case-control research, administered the Woodcock-Johnson IV cognitive and achievement test [24] remotely or in the face-to-face condition to two groups of 120 participants each. This test is used to evaluate intellectual abilities, on the one hand, and reading, writing, and math skills, on the other. No significant differences emerged between the performances obtained with the two different assessment modalities.

The same author, comparing teleassessment and face-to-face performance in the Reynolds Intelligence Assessment Scale [25], did not find significant differences in most of the subtests. However, in the case of the Processing Speed ​​Index, an assessment-method-related effect was found, particularly in subjects under the age of seven. Participants in the remote administration condition scored approximately seven standard score points lower on the Processing Speed ​​Index and approximately three points lower on the total score [26]. Similarly, the comparison of groups of children who took the Wechsler Intelligence Scale for Children V test [27] in teleassessment or face-to-face modality led to the identification of only a few differences.

Nevertheless, better performance in the face-to-face modality was found, specifically in the Letters-Numbers Reordering test [28]. Hamner et al. [29] administered the same intelligence scale and the Kaufman Test of Educational Achievement and confirmed the substantial equivalence in most of the subtests; however, unlike Wright [26], they obtained better results in teleassessment, compared to face-to-face administration, in the visual puzzle task of the WISC-V and in the Math Concept of the Kaufman test. Data from these cross-sectional studies suggest that the performance obtained in teleassessment could be considered comparable to that in face-to-face administration modality. However, some differences emerged showing that there is not always a full overlap between the two assessment modalities, leading to the need for further research in this field [13].

### Teleassessment of language and learning ability

Studies regarding receptive and expressive language in samples of children (up to school-aged children) report a good level of equivalence between the two assessment modalities, but in some cases, small groups of participants are tested [30]; in other studies, participants are evaluated in a single testing session, with an experimenter in the same room and another experimenter connected remotely [31]. Raman et al. [32] obtained similar results involving an active facilitator present in the school, who interacted with the child and played a crucial role in overcoming motivation and modulating the interaction with the online experimenter. By testing language comprehension abilities with the Peabody Picture Vocabulary Test, Fourth Edition [33], researchers found a non-complete equivalence between the in-person and teleassessment versions of the test [11].

With respect to receptive language skills, reading comprehension skills have also been tested. It has been observed that comprehension of materials could be affected by the medium used (paper or screen) and presentation modality (e.g., text, video, or subtitled video) [34, 35]. Text comprehension performance in paper-and-pencil assessment was substantially higher compared to performance mediated by electronic devices (e.g., computers, tablets, mobile phones, and e-readers) [36–38]. It has been shown that primary school children achieve lower scores on digital tests than on paper tests [35, 39, 40]. Feeling comfortable with the device used during the assessment could influence the results [41–43].

Similarly, in the learning domain, some studies demonstrate that the use of different media and of the teleassessment modality could negatively influence the observed performance. The Program for International Student Assessment [44] evaluated the skills of 15-year-old students in the fields of mathematics, reading, and science. Changes applied in the assessment modality—from paper-based tests to computer-based tests—negatively affect performance in all these abilities [45, 46].

A comparison of computerised and paper instruments for math and reading abilities often shows similar results in terms of outcomes in primary school children [47–49]. Nevertheless, this evidence has been obtained with cross-sectional studies or by comparing the performance obtained in a single session and evaluated by two different experimenters, one online and one physically present [14, 50].

Some authors noticed that, when tasks are administered in counterbalanced order (i.e., half of the participants complete the two conditions in one order and the other half complete the conditions in the reverse order), paper-and-pencil performance results can be better than those obtained in computer-administered tests [51]. Bergstrom noted that, in multiple domains (e.g., biology and reading), performance was influenced by administration modality, in favour of paper-and-pencil tests. Effectiveness in mastering test topics (e.g., reading, writing, and solving calculations) and effectiveness in mastering a situation (face-to-face or computer assessment modality) are largely mediated by one’s previous experience and could affect performance, as described in studies on the effects of computer experience [52–54]. Previous experience with both online and face-to-face learning modalities produces stronger outcomes with moderate effect size compared with experience with a single modality [55].

Research on learning domains has shown that teleassessment and in-person evaluations can be comparable, even if some differences emerge [14]. For example, Petrill et al. [56] showed that, in evaluating reading and mathematics abilities, correlations between the two administration modalities are reliable and valid (ranging between 0.52 and 0.92) but not completely equivalent.

Interestingly, Harder et al. [57], considering the two administration modalities, reported an order effect in some of the tasks: those children who were initially tested via teleassessment showed greater improvement in the second evaluation compared to those who were initially evaluated in a face-to-face modality, suggesting difficulty for participants in the execution of tasks during teleassessment if they are not familiar with them. These data suggest that evaluating the possible order effect on performance mediated by the two administration conditions is necessary.

### Research hypotheses and objectives

The screening phase is a fundamental step to establish which children have reached an adequate level of preparation and which ones need more attention to reach adequate preparation and perhaps a diagnostic study for a possible diagnosis. Previous research has shown a possible influence of the administration method, with better performance in in-person evaluation [32, 51, 57]. Consequently, it becomes important to define whether face-to-face and online activities are equivalent assessment modalities.

The main objective of our research was to test the comparability between teleassessment and in-person evaluation in the learning domain. Second, we wished to address whether the order of administration of the two assessment modalities could influence performance. To this end, we administered learning tasks in a counterbalanced order, both in person and in teleassessment, to a sample of children attending the first two years of primary school.

## Methods

### Participants

We undertook a crossover randomised controlled trial design. Children were recruited from three schools in northern Italy. Five classes (first and second grade) participated in the project. Children were recruited randomly from classes to reach an equivalent number of boys and girls. All the children were Italian native speakers without any documented history of brain damage, hearing, or visual deficits. Children who had already received a diagnosis of a neurodevelopmental disorder or specific hearing, vision, or physical impairments were excluded from the research.

Sixty-four children (32 girls and 32 boys equally distributed in each class; mean age 7.5, SD = 0.70, range 6.2–9.4) attending the first (*n* = 32) and second (*n* = 32) years of primary school participated in the study. Evaluations were carried out in three primary schools in May 2021 and 2022.

A power analysis using the G*Power computer programme, constraining alpha to 0.05, showed that using a paired sample *t* test comparison, 52 participants were needed to detect a small significant effect (*d* = 0.35) with 80% power. Using a repeated-measures analysis of variance (ANOVA), 52 participants were needed to detect a small significant within-between interaction effect (*f* = 0.2) with 80% power.

### Instruments

Standardised tests used in clinical practice were administered. The same tests were performed twice (e.g., not alternate forms) to address practice effects. Articles and manuals describing reading, writing, and math tests have reported good levels of reliability and validity [58–64]; see the Supplementary Material). Due to the use of different items in the test in the different classes and the presence of multiple variables collected during the evaluations (speed, accuracy, and errors), performance was evaluated using standardised scores based on reference manuals. Performance in the different tasks was mediated to obtain a single composite *z* score for each domain: reading, writing and math. Starting from four measures for the reading domain, three measures for the writing domain, and five measures for the math domain, we obtained a single composite score for each domain and a total score. We then analysed these values to compare the face-to-face and teleassessment conditions.

### Reading domain: word and pseudoword reading abilities

Reading abilities were evaluated by administering word and pseudoword lists [58, 61, 62]. Two tasks were administered to evaluate reading skills. Examiners asked children to read word and pseudoword lists “as fast and accurately as possible.” Different lists were shown one at a time through the tablet screen (teleassessment) or in paper format (face-to-face). The word lists were composed of 30 words for first graders and 112 words for second graders. The pseudoword lists were composed of 30 pseudowords for first graders and 48 pseudowords for second graders. Time and errors were registered. Each measure was transformed into a *z* score and mediated into a composite score.

### Writing domain: text dictation and graphomotor fluency

Two tasks were administered to measure writing accuracy (dictation task) and writing speed (graphomotor fluency) [63].

In the writing accuracy test, the children were invited to write a short text, which was dictated aloud by the experimenter, on lined paper. The text was composed of 58 words for first graders and 81 words for second graders. Errors were registered and transformed into a *z* score.

In the writing speed test, the children were asked to write the two letters “le” in cursive font continuously and the numbers in letter in uppercase font on lined paper. The instructions were as follows: “Write as fast and as accurately as possible.” Children in both tasks wrote for one minute. The numbers of written graphemes were registered and transformed into a *z* score. From all the *z* scores, a single composite score was calculated.

### Math domain: mental calculation, written calculation, forward enumeration, number dictation, retrieval of numerical facts

To assess math abilities, we administered a standardised test [64]. To obtain a single *z* score representative of mathematical abilities, it was necessary to administer five subtests that evaluate mental calculation, written calculation, forward enumeration, number dictation, and the retrieval of numerical facts.

Mental calculation. The experimenter pronounced six operations, one at a time. The child was required to mentally solve each operation as quickly as possible (within a maximum time of 30 s). The execution time and accuracy were registered.

Written calculation. The experimenter pronounced two operations, one at a time. The child was required to write each operation on paper and to solve it as quickly as possible (within a maximum time of 60 s). The execution time and accuracy were registered.

Forward enumeration. Children were asked to count aloud in ascending order from 1 to 20 (for the first year of primary school) or to 50 (for the second year) as quickly and accurately as possible. The execution time and the total number of omissions/errors were registered.

Number Dictation. The experimenter pronounced eight numbers, one at a time, and the child was asked to write the number on paper. The number of errors was registered.

Retrieval of Numerical Facts. The experimenter pronounced six arithmetic operations, one at a time. The operation referred to numerical facts usually automated in the early stages of math learning (e.g., 4 + 4; 7 + 3). The child was required to mentally solve each operation as quickly as possible (within a maximum time of 5 s). If the child provided the correct answer but exceeded the time limit, the item was considered incorrect. The number of errors was registered.

### Procedure

The study took place in a quiet, well-lit room of each school involved in the project with a wireless 4G internet connection. Each child took part in two experimental sessions of approximately 30 min. In a counterbalanced order, two properly trained experimenters administered reading, writing, and math standardised tasks normally used to screen learning abilities at the end of the school year. At Time 1 (T1), each child, in a pseudorandomised order (*n* = 32 and *n* = 32), received the face-to-face assessment or the teleassessment first. The children were evaluated with the alternative modality after three weeks, at Time 2 (T2), to avoid the possibility that they could remember all the items of the different tasks and consequently answer mainly on the basis of previous answers.

An initial moment of the meeting, both in presence and in teleassessment evaluation, was always devoted to a brief talk with each child to introduce the examiner and the tasks and to let the child feel comfortable with the experimenter. In face-to-face assessment, the child was accompanied into the room by an assistant. The child sat at a table facing the experimenter, who directly administered the tasks using standardised instructions described in the manuals. In the case of teleassessment, the child sat at a table facing a tablet and was placed at approximately 60 cm. In front of the child, there was space left for a sheet (on one side with lines, on the other with spaces to enter the numbers of the calculation test). In teleassessment, a passive operator sat near the child; this presence was necessary inside the school, where young children could not be left alone. This person needed to be completely passive (to avoid interaction with the children during the evaluation) and could intervene only to solve technical problems or to provide furniture. In an ecological context, the passive operator could be a properly trained teacher, whereas in our research, it was a person on our staff and not from the school to ensure that his/her presence did not affect performance in any way.

The online experimenter could speak with the child via an online chat (Skype) and showed the materials of the various tasks on the screen of the tablet. The child was acquainted with the experimenters at the beginning of the teleassessment session; then, during the online administration, the experimenter turned off the camera and showed only the materials (or an inscription indicating the test area that was being evaluated: the reading, writing, or math task) until the end of the assessment. The child’s camera was always active, and the online administrator could always see the child and the paper. All the materials (i.e., word and pseudoword lists, examples of writing tasks, examples of math tasks) shown on the screen of the tablet perfectly matched the paper materials in terms of dimensions and quality. To guarantee the same quality of the face-to-face and teleassessment evaluations, the third experimenter recorded the entire session, and the online experimenter could then check the accuracy of the response time and errors.

### Data analysis

To evaluate the validity of the two assessments, we conducted correlations between scores at the two administration times. To evaluate the effect of assessment type, we used a *t* test to directly compare performances considering the three domains together and then each domain separately. To evaluate the effect of assessment type and its interaction with previous experience with administered tests, we analysed data in the three domains in the two counterbalanced assessment conditions using mixed design analysis of covariance (ANCOVA), adding school grade as a covariate. We used pairwise comparisons with Bonferroni correction to evaluate significant interactions. To evaluate the dispersion and shape of the scores in the two administrations, we used a Kolmogorov–Smirnov analysis.

## Results

### Direct comparison of face-to-face and teleassessment evaluations

The overall performance results of the face-to-face and teleassessment evaluations were strongly related (*r* =.87). Furthermore, reading (*r* =.89), writing (*r* =.82), and math (*r* =.76, all *p*s > 0.001) evaluations were strongly related.

Kolmogorov–Smirnov analysis revealed that, when comparing groups at T1 and T2, the distribution shape of performance was not equivalent at T1 (Kolmogorov–Smirnov *z* = 1.625, *p* =.01) and was limited to the math domain, whereas it was equivalent at T2 (Kolmogorov–Smirnov *z* = 0.625, *p* =.830). The global distributions and the reading and writing domains appear to be equivalent at both T1 and T2 (all *p*s > 0.158).

**Fig. 1:**
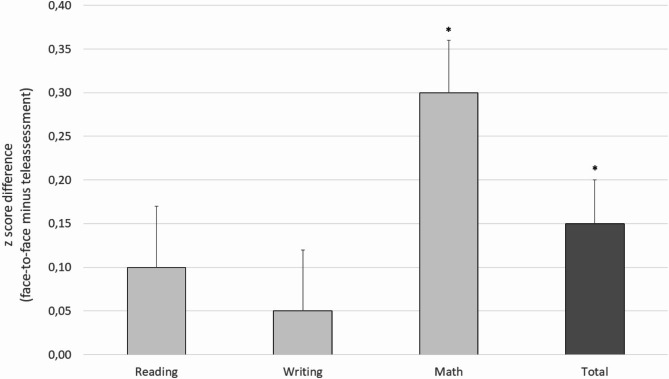
Delta score (z score difference) between face-to-face and teleassessment performance. Asterisks indicate a significant difference between the two modalities; bars represent the standard error of the mean

A direct comparison using a paired *t* test revealed that the overall performance result in the face-to-face modality (*z* score mean=-0.47, *SD* = 0.71) was greater than that in the teleassessment modality (*z* score mean=-0.63, *SD* = 0.80, *t*_(63)_ = − 2.916, *p* =.005, Cohen’s *d* = 0.36). Paired *t* tests on each of the three domains revealed that a significant difference (*t*_(63)_ = − 4.686, *p* <.001, Cohen’s *d* = 0.59) was present in the math domain (see also Table 1; Fig. 1, and Table 1S).

**Table 1:** *Z* score means and standard deviations (in parentheses) for the two evaluation modalities

	Reading z score	Writing z score	Math z score	Total z score
Face-to-face	−0.25 (0.99)	−1.38 (0.98)	0.21 (0.67)	−0.47 (0.71)
Teleassessment	−0.35 (1.10)	−1.44 (1.07)	−0.09 (0.77)	−0.63 (0.81)
*p* value and Cohen’s *d*	*p* =.168 *d* = 0.17	*p* =.42 *d* = 0.10	*p* <.001* *d* = 0.59	*p* =.005* *d* = 0.36

Asterisks indicate a significant difference between face-to-face and teleassessment performance (*p* <.05)

### Measurement of the effects of the order of face-to-face and teleassessment evaluation

To investigate the possible effects of the order of the two kinds of evaluation, we performed a mixed ANCOVA with domain (reading, writing, math tasks) and time of evaluation (T1 and T2) as within factors and evaluation order (face-to-face/teleassessment, teleassessment/face-to-face) as a between factor. The school year (first or second) was entered as a covariate. The mean *z* scores obtained in the three learning domains (i.e., reading, writing, and math) were used as dependent variables.

The main effects of school year (*F*_(1,61)_ = 17.739, *p* <.001, η^2^_p_ = 0.195) and the school year*task domain interaction were significant (*F*_(1,122)_ = 5.935, *p* =.003, η^2^_p_ = 0.003). See the Supplementary Material, other interactions of the covariate were significant.

The main effect of the task domain was significant (*F*_(2,122)_ = 29.507, *p* <.001, η^2^_p_ = 0.326; see Supplementary Material and Table 2).

**Table 2:** *Z* score mean and standard deviation (in parentheses) collapsing the two evaluation modalities

Assessment areas	z score mean
Reading ability	−0.30 (1.11)
Writing ability	−1.41 (0.99)
Math ability	0.06 (0.67)

**Fig. 2:**
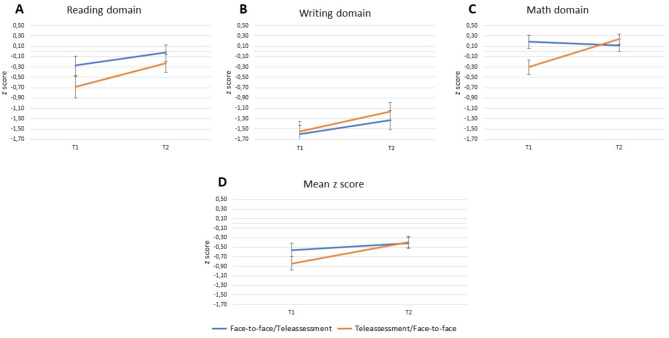
Mean performance of the two groups tested in the two different modalities in different orders

The main effect of time (*F*_(1,61)_ = 6.897, *p* =.011, η^2^_p_ = 0.102) and the time*evaluation order interaction were significant (*F*_(1,61)_ = 18.391, *p* <.001, η^2^_p_ = 0.232). Pairwise comparisons showed that the group that was initially evaluated in the face-to-face modality (*z* score mean = − 0.56, *SD* = 0.73) significantly improved its performance in the teleassessment evaluation at T2 (*z* score mean = − 0.41, *SD* = 0.72, *p* =.003, Cohen’s *d* = 0.65). Furthermore, the performance of the group initially evaluated via teleassessment (*z* score mean = − 0.84, *SD* = 0.85) significantly improved in the face-to-face evaluation at T2 (*z* score mean = − 0.38, *SD* = 0.69, *p* <.001, Cohen’s *d* = 1.45; see Fig. 2 Panel D).

The triple interaction task*time*evaluation order was significant (*F*_(1,122)_ = 5.787, *p* =.004, η^2^_p_ = 0.087). No other main effect or interaction was significant. When the class variable was excluded as a covariate, the main effects and interaction of the ANOVA remained unchanged.

To better understand the triple interaction, we conducted three different ANOVAs on the different task domains (reading, writing, and math abilities) with time of evaluation (T1 and T2) as within factors and evaluation order (face-to-face/teleassessment, teleassessment/face-to-face) as a between factor.

The ANOVA on *z* score performance in the reading domain showed that only the main effect of time (*F*_(1,62)_ = 36.501, *p* <.001, η^2^_p_ = 0.371) was significant. The time*evaluation order was only marginally significant (*F*_(1,62)_ = 3.079, *p* <.084, η^2^_p_ = 0.047). The main effect of the assessment order was not significant (see Fig. 2 Panel A and Table 3).

The ANOVA on *z* score performance in the writing domain revealed that only the main effect of time (*F*_(1,62)_ = 35.986, *p* <.001, η^2^_p_ = 0.367) was significant. No other main effect or interaction was significant (see Fig. 2 Panel B and Table 3).

The ANOVA on *z* score performance in the math domain showed that the main effects of time (*F*_(1,62)_ = 18.238, *p* <.001, η^2^_p_ = 0.227) and the time*evaluation order interaction were significant (*F*_(1,62)_ = 27.980, *p* <.001, η^2^_p_ = 0.311). As described in Table 3, pairwise comparisons revealed that the performance of the group that was evaluated initially in the face-to-face modality did not significantly improve in the teleassessment evaluation at T2 (*p* =.474). In contrast, the performance of the group that was initially evaluated via teleassessment significantly improved in the face-to-face evaluation at T2 (*p* <.001). Importantly, although the two groups were significantly different at T1 (*p* =.016, Cohen’s *d* = 0.62), the two groups did not differ at T2 (*p* =.983, Cohen’s *d*=-0.18). The main effect of the group variable was not significant (see Fig. 2 Panel A and Table 3).

The bars represent the standard error of the mean; Panel A shows performance in the reading domain; Panel B shows performance in the writing domain; Panel C shows performance in the math domain; and Panel D shows the total performance of the children in all three domains.

**Table 3:** Comparisons (p value and Cohen’s d) of performance at T1 and T2 according to administration order

Administration order	Reading			Writing			Math			Total		
	T1 mean (*SD*)	T2 mean (*SD*)	*p* value and Cohen’s *d*	T1 mean (*SD*)	T2 mean (*SD*)	*p* value and Cohen’s *d*	T1 mean (*SD*)	T2 mean (*SD*)	*p* value and Cohen’s *d*	T1 mean (*SD*)	T2 mean (*SD*)	*p* value and Cohen’s *d*
Face-to-face/teleassessment	−0.27 (0.99)	−0.02 (0.83)	*p* =.002 *d* = 0.60	−1.60 (0.94)	−1.33 (1.07)	*p* =.002 *d* = 0.60	0.19 (0.75)	0.12 (0.70)	*p* =.50 *d* = − 0.12	−0.56 (0.73)	−0.41 (0.72)	*p* =.003 *d* = 0.65
Teleassessment/face-to-face	−0.68 (-0.25)	−0.23 (0.98)	*p* <.001 *d* = 0.89	−1.55 (1.07)	−1.16 (0.98)	*p* <.001 *d* = 0.91	−0.30 (0.79)	0.24 (0.59)	*p* <.001 *d* = 1.27	−0.84 (0.85)	−0.38 (0.69)	*p* <.001 *d* = 1.45

## Discussion

The main objective of our research was to test whether in-person evaluation of the learning skills of primary school children can be considered equivalent to teleassessment performance. In the literature, the two modalities seem to be equivalent, despite some research demonstrating that teleassessment evaluations could lead to worse performance. In our study, the teleassessment scores were lower than the face-to-face ones; math tests weighted the most on difference. The novel feature of this research lies in not having evaluated a single aspect of learning but in the use of a complete battery of neuropsychological tests that are usually administered in the screening and evaluation phases of learning disability.

Together with the total performance, it is important to observe that the assessment modality effects, with values between negligible and medium, appeared to always be in the direction of better performance in the face-to-face condition. The performance of the two modalities was strongly correlated, indicating good reliability of the tests. In the math domain, however, performance distribution shape in the face-to-face and teleassessment modalities became equivalent only at T2, probably indicating a link with previous experience with tests and, overall, an effect of familiarity with the tasks [51–54, 65]. This could indicate that the two kinds of assessment can be considered equivalent [47], ensuring only prior familiarisation with the tasks [51, 57]. As described in a review by Ruffini et al. [14], despite a high level of reliability, the administration modalities, especially in the learning domain, did not show perfect overlap. In our research, we found good similarities between performances, but we also found some differences that could be highlighted in connection with the adequate sample size. Contrary to our expectation, the effects were related mainly to the math domain. It is possible to hypothesise that differences in math tasks could be explained by the domain itself; moreover, math skills in the first two years of primary school are the least trained abilities in the Italian school context compared to reading and writing skills [66]. It may also be possible to hypothesise that the performance difference could be linked to the complexity of the tests. While reading and writing tests evaluate the automatisation level of different learning domains, math tests require greater involvement of working memory and information processing [67, 68]. Consequently, it is possible that in this kind of task, as observed in semantic skill tasks, it is easier to obtain different performances related to administration conditions, especially in time-constrained tests [69–71].

A difference in performance in math tasks has not been found in research that compares the use of paper and pencil or computers [48, 49]. In this sense, we could hypothesise that the observed difference may not be linked to the medium per se but rather to the higher level of complexity of interaction between the examiner and the participant in the teleassessment modality.

As a second aim, in our research, we wished to address whether the order of administration of the two assessment modalities could influence performance. The results of the ANCOVA and subsequent analysis in a single domain showed that, together with a test–retest effect, it is possible to observe a stronger improvement at T2 in the group tested in the teleassessment modality at T1. Math tests are crucial for highlighting this type of effect. In this domain, improvements between T1 and T2 doubled (Cohen’s *d* = 1.27) compared to differences between T1 and T2 in the group that was initially tested in a face-to-face modality (Cohen’s *d* = 0.12). Importantly, in the first assessment session, a difference between the two evaluation modalities was observed: Children assessed in the face-to-face modality performed better than children tested in the teleassessment modality did (Cohen’s *d* = 0.62). When the evaluation methods were reversed in the second session, the differences between the two groups disappeared: the performance of the children initially evaluated with teleassessment improved, becoming equivalent to that of the children initially evaluated in the face-to-face condition (Cohen’s *d* = − 0.18). This result seems to confirm that although a test–retest effect was observed, the teleassessment condition seemed to be linked to greater difficulty in performing the task. In the math domain, in which differences appear to be easier to find, the test–retest effect is nullified in the group initially assessed in the face-to-face condition, indicating difficulty in performing these tasks in the teleassessment modality.

These results agree with previous research by Harder et al. [57], where an order effect was found in some of the tasks. As described in the introduction, in their study, the children who were initially tested via teleassessment showed greater improvement in the second evaluation than did the children who were initially evaluated in the face-to-face modality. Our data seem to confirm difficulty for children in the execution of tasks during teleassessment if they are not familiar with them. These data suggest that evaluating a possible order effect on performance mediated by the two administration modalities is necessary.

Notably, during our administration, as in most research on children, a passive experimenter was present inside the room to guarantee the availability of materials (e.g., paper) and the perfect functioning of the internet connection. The presence in the room of a passive researcher guaranteed that the children’s performance was genuine. In this sense, other research shows the crucial role that an active administrator could play in maintaining attention, overcoming motivation problems, and modulating interaction during teleassessment [32], confirming that although teleassessment could be considered a motivating environment [14], online evaluation can lead to lower performance.

## Conclusions

Due to restrictions related to the COVID-19 pandemic, teleassessment modalities have become widespread. Although comparability between face-to-face and remote assessment has not yet been established [14], in our research, we wanted to investigate the effects of using an online assessment modality compared with traditional methods.

We showed that performance in face-to-face and teleassessment evaluations is not completely comparable. Worse performance was observed in the teleassessment. The administration order of modalities indicates that previous experience with materials during face-to-face administration facilitates execution in teleassessment. Differences in the math domain mainly drove the observed differences.

A limitation of our study is the absence of data about socioeconomic status, which could influence learning performance and experience with electronic devices [17]. Future research should also include measurements of specific individual characteristics, such as self-efficacy, that could influence the difference between face-to-face and teleassessment performance [72].

Notably, our sample of children, independent of the evaluation modality, shows a normal but relatively low level of performance in multiple domains. Although this could be attributed to characteristics randomly present in the population, the low level reached by the children in standardised tasks could be linked to the multiple suspensions of school activities because of pandemic restrictions [73–75].

Our results show that teleassessment could lead to an overestimation of children at risk for learning disorders. Future research could investigate whether the observed differences could be linked to specific domains or to specific cognitive functions involved in task execution.

## Electronic supplementary material

Below is the link to the electronic supplementary material.

Supplementary Material 1

## Data Availability

The datasets used and/or analysed during the current study are available from the corresponding author on reasonable request.

## Abbreviations

ANCOVA: Analysis of covariance
ANOVA: Analysis of variance
T1: Time 1
T2: Time 2
