# Supplementary material for: Teleassessment can overestimate the risk of learning disability in first and second grade of primary school
Source: Ital J Pediatr. 2025 Feb 11;51:40. doi: 10.1186/s13052-025-01881-4 (PMC11817320; doi:10.1186/s13052-025-01881-4)
Supplement: Supplementary file 1 — Supplementary Material 1 [file 13052_2025_1881_MOESM1_ESM.docx]

**Supplementary Material**

**Instruments: Reliability and validity**

Articles and manuals describing the different tests for the evaluation of reading, writing and math abilities report good levels of reliability and validity.

About reading tests, analysis comparing tests administered in our research show good levels of validity. Between De.Co.Ne. and DDE-2 tests, a correlation of r=.88 for word reading and r=.83 for pseudoword reading is reported, showing a high correlation between the two tests. The validity of pseudoword reading test in De.Co.Ne. battery is also confirmed by comparing it with different reading tests (other pseudoword list, pseudoword text) present in the same battery, showing a correlation of .88 and .82, respectively. Reading performances in DDE-2 test have been compared to different Italian tests [59]. Word lists of DDE-2 resulted related to the other word lists (r=.95 high frequency word; r=.96 low frequency word). Similar results were observed about correlation between the pseuodoword lists (r=.96). About the reliability for the DDE-test, a test-retest procedure was assessed during the implementation of the test. Seventy-seven subjects repeated the test 20 days after the first administration, showing a good correlation between reading time scores (r= .77) and reading error measurements (r=.56).

About writing abilities, fluency writing tests in the BVSCO-2 battery showed a good reliability (r=.64) in a test-retest analysis assessed at least two months of distance (hundreds of children were evaluated). About the validity, Authors report a correlation between writing speed and text copying skills (r=.73).

The writing/reading Martini test shows a correlation with other reading/writing tests (r=.78, r=.81 administered at different times of the school year) [60].

About math skills, AC-MT Authors report that all subtests have a good reliability (α>.5). Test-retest correlations, at several months, is equal to r=.73.

**Results**

Mixed analysis of covariance (ANCOVA) results:

The main effect of school grade (F_(1,61)_=17.739, p<.001, η^2^_p_=.195) and school year and task domain interaction were significant (F_(1,122)_=5.935, p=.003, η^2^_p_=.003). Pairwise comparisons showed that performance in reading (mean=-.60, SD=.93) and in writing (mean=-1.92, SD=.64) domains of children attending the first year were significantly lower than those of children at the second year (reading mean=.00, SD=.99; writing mean=-.89, SD=1.01 and p<.001 respectively). No other interaction of the covariate was significant.

The main effect of the task domain was significant (F_(2,122)_=29.507, p<.001, η^2^_p_=.326). Pairwise comparisons, controlling for school grade, showed that performance in math tasks were significantly higher than in reading (p=.009) and writing tasks (p<.001), and performance in reading tasks were significantly higher (p<.001) than in writing tasks.

**Table 1S.** Raw scores (mean and standard deviation) in different tasks, divided for classes and administration modality.

|  | First grade of primary school | | Second grade of primary school | |
| --- | --- | --- | --- | --- |
|  | Face-to-face  Mean (Standard deviation) | Teleassessment  Mean (Standard deviation) | Face-to-face Mean (Standard deviation) | Teleassessment  Mean (Standard deviation) |
|  |  |  |  |  |
| Word reading (syll/sec) | 1.03 (0.66) | 0.94 (0.50) | 1.96 (0.77) | 1.92 (0.74) |
| Word reading (errors) | 3.97 (3.56) | 3.41 (3.43) | 8.72 (7.43) | 8.69 (6.89) |
| Pseudoword reading (syll/sec) | 1.06 (0.43) | 0.94 (0.41) | 1.32 (0.46) | 1.26 (0.41) |
| Pseudoword reading (errors) | 8.00 (5.05) | 8.34 (4.47) | 8.28 (5.60) | 8.28 (6.42) |
| Writing - text dictation (errors) | 20.69 (6.18) | 21.41 (6.84) | 9.25 (6.54) | 10.91 (7.07) |
| “Le” writing (numbers of graphemes) | 29.53 (9.40) | 30.88 (10.48) | 51.06 (9.72) | 51.81 (13.77) |
| “Numbers” writing (numbers of graphemes) | 29.13 (10.97) | 29.41 (10.88) | 66.09 (12.38) | 67.53 (14.72) |
| Math - mental calculation (errors) | 1.78 (1.39) | 1.53 (1.34) | 0.72 (0.89) | 0.66 (1.10) |
| Math - mental calculation (sec) | 47.66 (26.27) | 56.06 (33.69) | 28.94 (12.18) | 35.00 (15.17) |
| Math - written calculation (errors) | 0.28 (0.52) | 0.34 (0.60) | 0.22 (0.42) | 0.22 (0.42) |
| Math - written calculation (sec) | 19.69 (11.33) | 27.50 (17.07) | 30.31 (12.13) | 38.69 (17.78) |
| Math - Forward enumeration (errors) | 0.03 (0.18) | 0.22 (0.55) | 0.06 (0.25) | 0.03 (0.18) |
| Math - Forward enumeration (sec) | 9.28 (2.20) | 10.53 (2.36) | 29.47 (6.49) | 31.56 (6.36) |
| Math - Number dictation (errors) | 0.13 (0.34) | 0.28 (0.58) | 0.03 (0.18) | 0.00 (0.00) |
| Math - Retrieval of numerical facts (errors) | 1.81 (1.57) | 2.63 (1.58) | 0.50 (0.84) | 1.06 (1.27) |
